# Adsorption behaviors and mechanisms of Cu^2+^, Zn^2+^ and Pb^2+^ by magnetically modified lignite

**DOI:** 10.1038/s41598-022-05453-y

**Published:** 2022-01-26

**Authors:** Junzhen Di, Zhen Ruan, Siyi Zhang, Yanrong Dong, Saiou Fu, Hanzhe Li, Guoliang Jiang

**Affiliations:** School of Civil Engineering, Liaoning Technical University, Fuxin, 123000 China

**Keywords:** Environmental sciences, Environmental chemistry

## Abstract

The study aims to solve the problems of limited capacity and difficult recovery of lignite to adsort Cu^2+^, Zn^2+^ and Pb^2+^ in acid mine wastewater (AMD). Magnetically modified lignite (MML) was prepared by the chemical co-precipitation method. Static beaker experiments and dynamic continuous column experiments were set up to explore the adsorption properties of Cu^2+^, Zn^2+^ and Pb^2+^ by lignite and MML. Lignite and MML before and after the adsorption of heavy metal ions were characterized by scanning electron microscopy (SEM), X-ray diffraction (XRD) and Fourier transform infrared spectrometer (FTIR). Meanwhile, the adsorption mechanisms of Cu^2+^, Zn^2+^ and Pb^2+^ by lignite and MML were revealed by combining the adsorption isotherm model and the adsorption kinetics model. The results showed that the pH, adsorbent dosage, temperature, initial concentration of heavy metal ions, and contact time had an influence on the adsorption of Cu^2+^, Zn^2+^ and Pb^2+^ by lignite and MML, and the adsorption processes were more in line with the Langmuir model. The adsorption kinetics experiments showed that the adsorption processes were jointly controlled by multiple adsorption stages. The adsorption of heavy metal ions by lignite obeyed the Quasi first-order kinetic model, while the adsorption of MML was chemisorption that obeyed the Quasi second-order kinetic model. The negative Δ*G* and positive Δ*H* of Cu^2+^ and Zn^2+^ indicated the spontaneous and endothermic nature reaction, while the negative Δ*H* of Pb^2+^ indicated the exothermic nature reaction. The dynamic continuous column experiments showed that the average removal rates of Cu^2+^, Zn^2+^ and Pb^2+^ by lignite were 78.00, 76.97 and 78.65%, respectively, and those of heavy metal ions by MML were 82.83, 81.57 and 83.50%, respectively. Compared with lignite, the adsorption effect of MML was better. As shown by SEM, XRD and FTIR tests, Fe_3_O_4_ was successfully loaded on the surface of lignite during the magnetic modification, which made the surface morphology of lignite coarser. Lignite and MML removed Cu^2+^, Zn^2+^ and Pb^2+^ from AMD in different forms. In addition, the adsorption process of MML is related to the O–H stretching vibration of carboxylic acid ions and the Fe–O stretching vibration of Fe_3_O_4_ particles.

## Introduction

In the process of coal mining, the original reduction environment can be changed into oxidation environment. Under the action of bacteria and oxygen, the existing sulfide can produce a large number of acid substances, which are dissolved in water and to form acid mine wastewater (AMD)^1^. AMD has multiple hazards to the natural environment, embodied in low pH value, high sulfate concentration and heavy metal ion content^2^. Among them, heavy metal ions such as Cu^2+^, Zn^2+^, and Pb^2+^ cannot be biodegraded or metabolized. After a series of food chain conduction, these heavy metal ions can be easily ingested into human body, and causing many health threat^3,4^. Therefore, it is necessary to find some reliable methods to remove heavy metal ions such as Cu^2+^ Zn^2+^, and Pb^2+^ from AMD.

The treatment methods of heavy metal pollution in AMD mainly include chemical precipitation, microbial, wetland and adsorption method^5–8^. Among them, adsorption method has the advantages of simple operation and treatment efficiency, which is widely used in the field of water treatment^9,10^. Zendelska et al.^11^ treated Zn^2+^ in AMD with zeolite-bearing tuff (stilbite) and analyzed the influence of each factor on the treatment effect through single factor experiment. Lin et al.^12^ showed that spent shiitake substrate could be used to adsorb copper ions in AMD. Yang et al.^13^ used modiied pyrite to conduct batch and column experiments to study its adsorption capacity for copper in simulated and actual AMD. Lin et al.^14^ used coffee grounds as adsorbent to adsorb Pb^2+^ and Zn^2+^ in AMD. The results showed that the maximum adsorption capacity of coffee grounds for Pb^2+^ and Zn^2+^ was 5.49 and 12.38 mg/g, respectively. At present, there are a variety of adsorption materials used to treat heavy metal ions in AMD, but there are problems of high treatment dosage and cost. Therefore, looking for an economical and reliable adsorption material has become a hot topic in the current field.

Lignite is rich in resources and low in price, mainly used in power plant fuel. However, lignite combustion seriously pollutes the air environment, which limits its use to a certain extent. Mohan et al.^15^ confirmed that lignite can be used as an adsorbent, which is rich in humic acid and has oxygen-producing functional groups such as carboxyl, hydroxyl and methoxyl, and has certain adsorption effect on heavy metal ions^16,17^. Jellali et al.^18^ showed that lignite could remove cadmium and copper from aqueous solution under static experimental conditions, and the removal of cadmium accounted for 78% of the total adsorption capacity within one minute. Munir et al.^19^ applied bamboo biochar and lignite together, and explored the effect of bamboo biochar/lignite on the removal of copper ions in pore water by comparing the uptake of copper ions by rapeseed and wheat before and after application. However, the primary lignite has complex composition, single void structure and limited adsorption capacity for heavy metal ions. In order to improve the adsorption performance of lignite, many scholars have modified lignite. Sakthivel et al.^20^ used facile depolymerization and Friedel Craft’s alkylation to improve the wettability of lignite, and the removal rate of Cr(VI) of chemically modified lignite was still as high as 90–95% after 4–5 desorption tests. Regassa et al.^21^ treated lignite with acid, and the removal rate of Cr(VI) from acid-modified lignite could reach 98% under certain conditions. He et al.^22^ prepared copper-containing adsorbents by ultrasonic impregnation protocol and lignite as precursor, and calculated by Langmuir isothermal model that the maximum adsorption capacity of direct yellow brown D3G in wastewater at 25 °C was 369 mg/g. These studies further confirm the potential of lignite in the field of adsorption.

Although lignite can be modified to improve its adsorption performance, it is difficult to precipitate and separate lignite in wastewater treatment due to its suspension. Cheng et al.^23^ used chitosan as bridging reagent to prepare magnetic Fe_3_O_4_ particle modified sawdust, which can be quickly separated from the solution and has a maximum adsorption capacity of 12.59 mg/g for strontium ions in the solution. The magnetic natural composite Fe_3_O_4_-chitosan@bentonite synthesized by Feng et al.^24^ can be easily recovered by external magnetic field after AMD treatment. Moreover, the adsorption capacity of Cr(VI) decreased only 3% after five consecutive adsorption–desorption processes. Chen et al.^25^ modified attapulgite with hydrochloric acid and mixed it with Fe_3_O_4_ to prepare adsorption material for Cr(VI) treatment in water. The study shows that the removal rate of Cr(VI) is as high as 95% within 5 min, and it can be easily removed from aqueous solution by external magnetic field after treatment. The above researches show that loading magnetic source of Fe_3_O_4_ onto the surface of solid particles can not only solve the problem of difficult separation of powder adsorbent with large specific surface area which from the target solution, but also improve the adsorption performance of the adsorbent. Based on the above considerations, functionalization of lignite with Fe_3_O_4_ can be considered for the removal of heavy metal ions in AMD.

In this paper, magnetically modified lignite (MML) was prepared by chemical co-precipitation method and used to remove Cu^2+^, Zn^2+^ and Pb^2+^ from AMD. The removal effects of Cu^2+^, Zn^2+^ and Pb^2+^ by Lignite and MML in AMD were compared by static beaker experiments and dynamic continuous column experiments, and the lignite and MML materials before and after the adsorption of heavy metal ions were characterized by SEM, XRD and FTIR. At the same time, the adsorption mechanisms of Cu^2+^, Zn^2+^ and Pb^2+^ by lignite and MML were revealed by combining the adsorption isotherm model and the adsorption kinetics model. Through this study, it can not only solve the problem that lignite is difficult to separate from solution, but also improve the ability of lignite to adsorb heavy metal ions. The treatment of AMD has significant environmental, social and economic benefits.

## Results and discussion

### Effect of pH, adsorbent dose and temperature

#### Effect of pH

**Figure 1:**
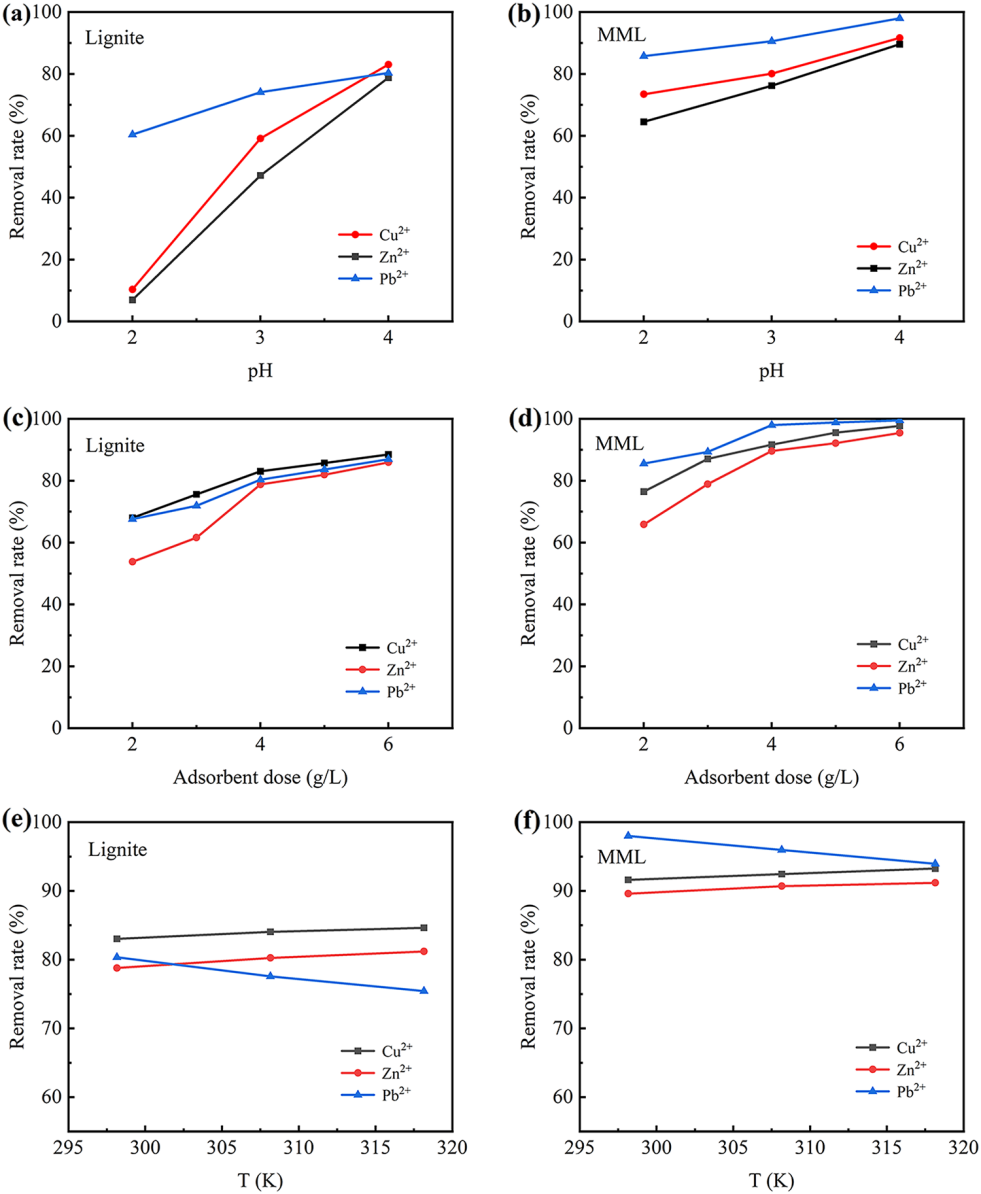
Relationship between the pH value and adsorption effect: (**a**) lignite; (**b**) MML. Relationship between adsorbent dose and adsorption effect: (**c**) lignite; (**d**) MML. Relationship between temperature and adsorption effect: (**e**) lignite; (**f**) MML.

According to the actual pH of AMD, the effects of different pH (2–4) on the adsorption process of Cu^2+^, Zn^2+^ and Pb^2+^ by lignite and MML were studied as shown in Fig. 1a,b. With the increase of pH value, the adsorption of Cu^2+^, Zn^2+^ and Pb^2+^ by lignite and MML gradually increases. When pH = 4, the removal rates of heavy metal ions reaches the maximum. Among them, the removal rates of Cu^2+^, Zn^2+^ and Pb^2+^ by lignite are 83.02, 78.79 and 80.34%, respectively, and those of heavy metal ions by MML are 91.61, 89.60 and 98.00%, respectively. It can be seen that MML has higher adsorption capacity than lignite. In addition, the results show that pH has a great influence on the adsorption process. Because large amount of H^+^ in a strong acidic solution would compete with heavy metal cations for adsorption sites, leading to the low removal rates under lower pH value. Therefore, the optimum pH value is 4.

#### Effect of adsorbent dose

The effects of different adsorbent dosage on the adsorption process of Cu^2+^, Zn^2+^ and Pb^2+^ were studied as shown in Fig. 1c,d. It can be seen that with the increase of adsorbent amount, the removal rates also increases. For MML, when the adsorbent amount is 4 g/L, the removal rates of heavy metal ions exceeds 89%. When the amount of MML is increased to 6 g/L, the reaction tends to be balanced, and the removal rates is not significantly improved. Therefore, considering the adsorption effect and economic cost, the optimum adsorbent amount is 1 g/L.

#### Effect of temperature

Temperature is an important parameter in the adsorption process. The effects of temperature on the adsorption process of heavy metal ions at different temperatures (298.15, 308.15 and 318.15 K) were shown in Fig. 1e,f. The removal rates of Cu^2+^ and Zn^2+^ by lignite and MML increases with the increase of temperature, indicating that the adsorption process is endothermic. However, the removal rate of Pb^2+^ decreases with the increase of temperature, which indicates that the adsorption process of Pb^2+^ by lignite and MML is exothermic. Although the removal rates of Cu^2+^ and Zn^2+^ by lignite and MML reaches the maximum at 318.15 K (i.e. 45 °C), most reaction systems are carried out at ambient temperature, so the optimum temperature is 298.15 K (i.e. 25 °C).

### Initial concentration and adsorption isotherm

#### Effect of initial concentration

**Figure 2:**
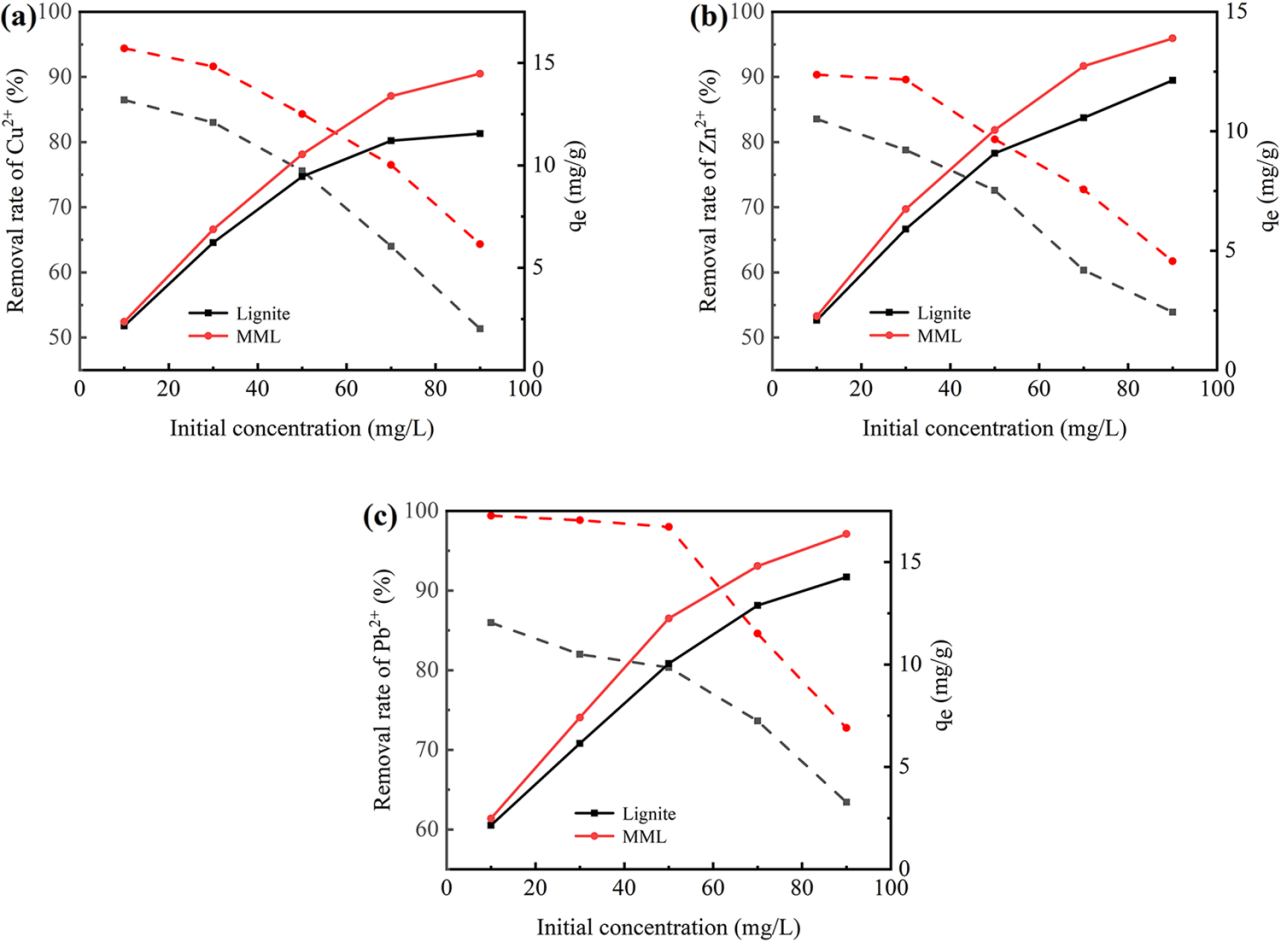
Relationship between initial concentration of heavy metal ions and adsorption effect: (**a**) Cu^2+^ removal effect; (**b**) Zn^2+^ removal effect; (**c**) Pb^2+^ removal effect.

The adsorption capacity and removal rates of lignite and MML for different initial concentrations of heavy metal ions were shown in Fig. 2. The adsorption capacity of heavy metal ions by lignite and MML increases with the increase of initial concentration.

This is because the higher the initial concentration of heavy metal ions, the higher the chance of collisions with adsorption sites on the surface of the adsorbent. Moreover, the driving force of mass transfer is better, which is conducive to reduce the mass transfer resistance and increase the adsorption capacity^26^. However, the removal rate of heavy metal ions by lignite and MML decreases with the increase of initial concentration. Especially when the initial concentration of Cu^2+^, Zn^2+^ and Pb^2+^ are 30, 30, and 50 mg/L respectively, the slope of the removal rate curve increases significantly. This is because for the fixed amount of adsorbent, the number of adsorption sites on the surface is limited, and the adsorption effect will achieve the best adsorption at a certain concentration of heavy metal ions.

Comparing the adsorption effects of lignite and MML on heavy metal ions, it can be seen that the adsorption capacity and removal rates of Cu^2+^, Zn^2+^ and Pb^2+^ by MML are higher than that of lignite at the same concentrated heavy metal ion degree. In the initial concentration range of 10–90 mg/L, the removal rates of Cu^2+^ and Zn^2+^ by lignite and MML shows similar trends, and the difference in removal rates tend to increase. In contrast, the removal rates of Pb^2+^ by MML in the range of 10–50 mg/L is almost constant with increasing initial concentration, indicating that Pb^2+^ in AMD is well removed by MML in this range. In addition, In the initial concentration range of 10–90 mg/L, the difference of adsorption of Cu^2+^, Zn^2+^ and Pb^2+^ between MML and lignite increases with the increase of initial concentration. This phenomenon indicates that MML has greater adsorption potential than lignite when the concentration of heavy metal ions in AMD solution is higher.

### Adsorption isotherm

The adsorption isotherm describes the relationship between the adsorbent and the amount of analytical substances in the solution^27^. To clarify the mechanism of Cu^2+^, Zn^2+^ and Pb^2+^ adsorption by lignite and MML, the Langmuir model and Freundlich model were used to fit the experimental data.

The Langmuir model assumes that monolayer adsorption occurs on the uniform adsorbent surface, with no interaction between adsorbates. The Langmuir model is expressed in the following form^28^:

1 $$\frac{{C}_{e}}{{q}_{e}}=\frac{{C}_{e}}{{q}_{m}}+\frac{1}{\left({K}_{L}{q}_{m}\right)}$$

where *q*_*m*_ and *q*_*e*_ are the maximum adsorption capacity and the adsorption capacity at equilibrium (mg/g), respectively, *C*_*e*_ is the adsorbate concentration in solution at equilibrium (mg/L), and *K*_*L*_ is the Langmuir adsorption constant (L/mg). The values of *q*_*m*_ and *K*_*L*_ can be calculated by a linear relationship. In addition, the equilibrium constant *R*_*L*_ of the Langmuir model can be used to describe the adsorption effect of the adsorption process. The *R*_*L*_ equation is of the following form^29^:

2 $${R}_{L}=\frac{1}{1+{K}_{L}{C}_{0}}$$

where *C*_*0*_ is the initial concentration of metal ions. The value *R*_*L*_ < 1 indicates good adsorption performance.

Based on multilayer adsorption on non-homogeneous surfaces, the empirical Freundlich equation without assumptions is expressed in the following form^30^:

3 $$\mathit{ln}{q}_{e}=\mathit{ln}{K}_{F}+\frac{1}{n}\mathit{ln}{C}_{e}$$

where *K*_*F*_ (L/mg) and *n* (dimensionless) are constant indications of the adsorption capacity.

**Figure 3:**
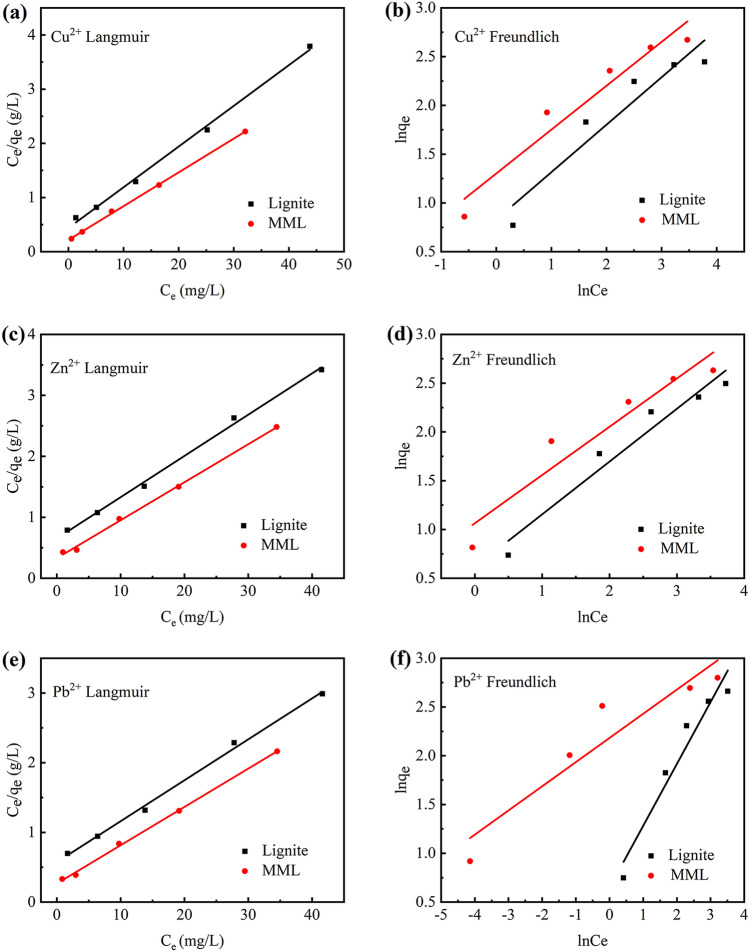
Linear plots of adsorption isotherms of heavy metal ion adsorption on different samples: lignite; MML. (**a**) Cu^2+^ Langmuir; (**b**) Cu^2+^ Freundlich; (**c**) Zn^2+^ Langmuir; (**d**) Zn^2+^ Freundlich; (**e**)Pb^2+^ Langmuir; (**f**) Pb^2+^ Freundlich.

The adsorption isotherms and corresponding parameters of Cu^2+^, Zn^2+^ and Pb^2+^ adsorption by lignite and MML were shown in Fig. 3 and Table 1, respectively. The correlation coefficient (*R*^*2*^ > 0.99) of the Langmuir model is higher, indicating that the processes of Cu^2+^, Zn^2+^ and Pb^2+^ adsorption by lignite and MML are more consistent with the Langmuir model (Table 1). On the basis of this result, it can be inferred that the processes of Cu^2+^, Zn^2+^ and Pb^2+^ adsorption by lignite and MML belong to Langmuir monolayer adsorption, where there is no interaction between the heavy metal ions adsorbed on the surface^31^. This indicates that a monolayer adsorbent is formed on the surface of lignite and MML, and no further adsorption will be carried out after the surface is completely covered^32,33^. In addition, the *R*_*L*_ value obtained according to Langmuir adsorption constant *K*_*L*_ is between 0 and 1, indicating that lignite and MML have good adsorption of heavy metal ions^34^. In Freundlich model, the 1/n value less than 1 also confirms that the adsorption conditions is good, and a smaller 1/n value indicates that MML is more favorable for the adsorption of Cu^2+^, Zn^2+^ and Pb^2+^ in the solution^35,36^.

**Table 1:** Adsorption isotherm constants for the adsorption of heavy metal ion onto different samples: lignite; MML.

Metal ion	Adsorption material	Langmuir				Freundlich		
		K_L_ (L/mg)	q_m_ (mg/g)	R^2^	R_L_	K_F_ (L/mg)	1/n	R^2^
Cu^2+^	Lignite	0.16684	13.36180	0.99576	0.16652	2.26204	0.48950	0.88550
	MML	0.25769	16.21270	0.99926	0.11458	3.47001	0.46680	0.91549
Zn^2+^	Lignite	0.10161	14.79290	0.99571	0.24702	1.82106	0.54410	0.93473
	MML	0.20573	15.80530	0.99772	0.13943	3.03035	0.47940	0.92316
Pb^2+^	Lignite	0.09121	17.52740	0.98246	0.17984	1.90594	0.63730	0.92816
	MML	0.25673	18.38700	0.99755	0.07227	8.85303	0.24820	0.85616

By comparing the parameters of lignite and MML adsorption isotherms, it can be seen that the Langmuir model of MML has a large correlation coefficient, which may be due to the uniform specific adsorption sites generated in the magnetization process^37^. It has been reported that the larger the adsorption capacity of the adsorbent, the greater the *K*_*F*_ value^38^. The *K*_*F*_ value of MML in this study is larger than that of original lignite, indicating that the adsorption capacity of MML is larger than that of lignite. The maximum adsorption capacity of MML for Cu^2+^, Zn^2+^ and Pb^2+^ are 16.2127, 15.8053 and 18.3870 mg/g, respectively, while the maximum adsorption capacities of lignite are13.3618, 14.7929 and 17.5274 mg/g, respectively. It is further confirmed that magnetic modification of lignite improves the adsorption capacity of Cu^2+^, Zn^2+^ and Pb^2+^.

### Contact time and adsorption kinetics

To clarify the adsorption mechanism of lignite and MML, the adsorption kinetics of Cu^2+^, Zn^2+^ and Pb^2+^ by lignite and MML were analyzed by Quasi first-order kinetic model, Quasi second-order kinetic model, Elovich model and Intra-particle diffusion model.

#### Quasi first-order model

Lagergren proposed an adsorption analysis method based on solid adsorption capacity^20^, which is the Quasi first-order kinetic equation in the following form^29,31^:

4 $$\mathit{ln}\left({q}_{e}-{q}_{t}\right)=\mathit{ln}{q}_{e}-{k}_{1}t$$

where *q*_*e*_ and *q*_*t*_ are the amounts of adsorbed metal ions at equilibrium and at time *t* (mg/g), respectively, and *k*_*1*_ is the Quasi first-order rate constant (min^−1^).

#### Quasi second-order model

The Quasi second-order kinetic model is based on the assumption that the adsorption rate is controlled by chemisorption^24^. The Quasi second-order kinetic model is expressed in the following form^39^:

5 $$\frac{t}{{q}_{t}}=\frac{1}{{k}_{2}{q}_{e}^{2}}+\frac{t}{{q}_{e}}$$

where *q*_*e*_ and *q*_*t*_ are the amounts of adsorbed metal ions at equilibrium and at time *t* (mg/g), respectively, and *k*_*2*_ is the Quasi second-order rate constant (min^−1^).

**Figure 4:**
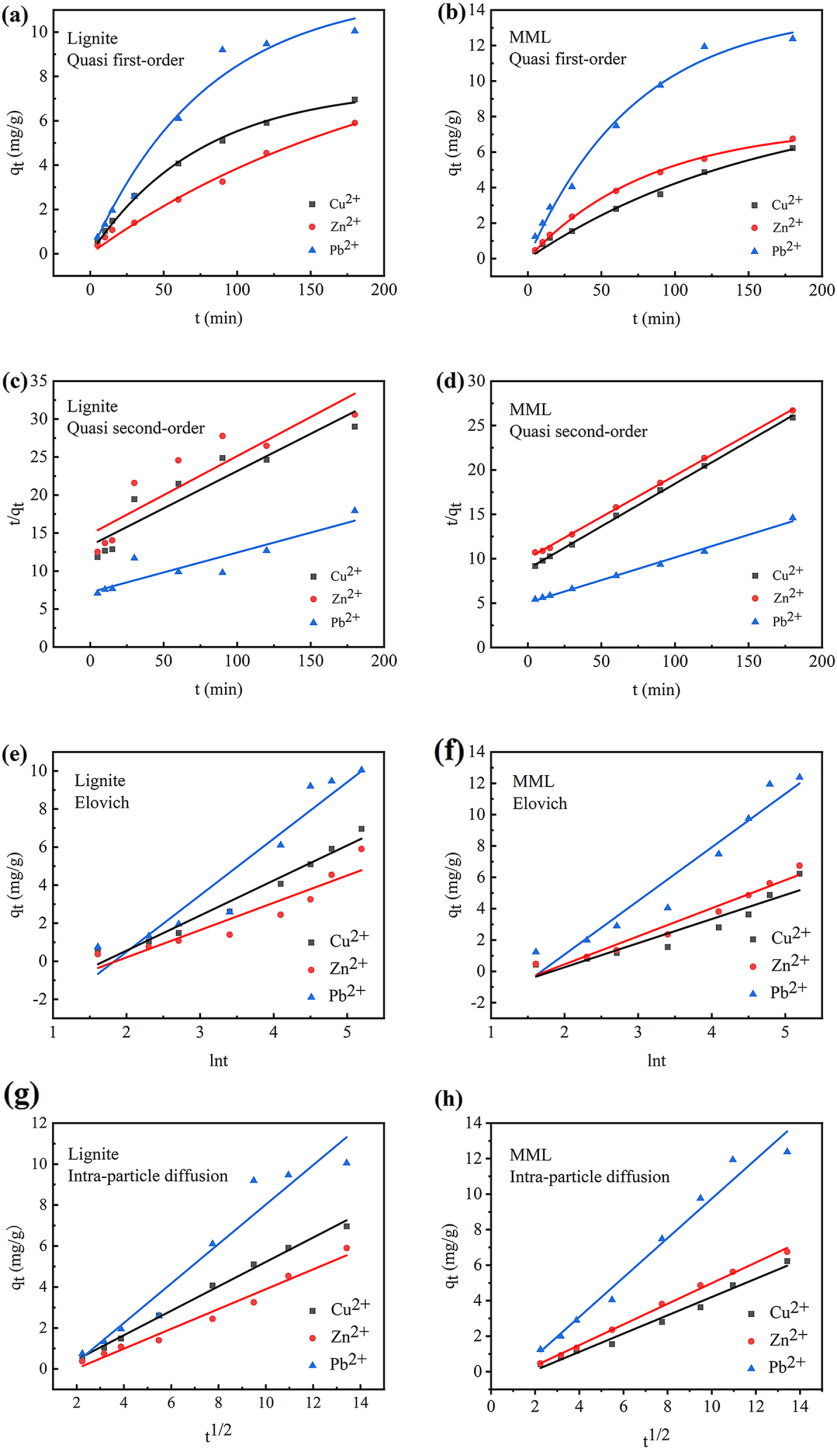
Adsorption kinetics for adsorption of Cu^2+^, Zn^2+^ and Pb^2+^ onto different samples: lignite; MML. (**a**) Quasi first-order kinetic model of the adsorption of lignite; (**b**) quasi first-order kinetic model of the adsorption of MML; (**c**) quasi second-order kinetic model of the adsorption of lignite; (**d**) quasi second-order kinetic model of the adsorption of MML; (**e**) Elovich model of the adsorption of lignite; (**f**) Elovich model of the adsorption of MML; (**g**) intra-particle diffusion model of the adsorption of lignite; (**h**) intra-particle diffusion model of the adsorption of MML.

The results of Quasi first-order model and Quasi-second-order kinetic fits for the adsorption of Cu^2+^, Zn^2+^ and Pb^2+^ by lignite and MML were shown in Fig. 4 and Table 2.

**Table 2:** Kinetic parameters of heavy metal ion adsorption on different samples: lignite; MML.

Metal ion	Adsorption material	Quas first-order model			Quasi second-order model			Elovich model			Intra-particle diffusion model		
		K_1_	q_e_ (mg/g)	R^2^	K_2_	q_e_ (mg/g)	R^2^	a	b	R^2^	K_3_	C	R^2^
Cu^2+^	Lignite	0.00607	9.26020	0.98854	0.00072	10.19260	0.85585	0.33849	0.54289	0.96605	0.73173	− 1.65400	0.87331
	MML	0.01311	7.53811	0.99724	0.00104	10.41780	0.99906	0.24709	0.65012	0.89240	0.87488	− 1.72723	0.95551
Zn^2+^	Lignite	0.00468	10.28394	0.98526	0.00070	9.74470	0.78164	0.22419	0.69708	0.87171	0.68009	− 1.56120	0.84320
	MML	0.01191	7.52736	0.99828	0.00087	10.71470	0.99925	0.31145	0.55851	0.95981	0.85380	− 1.78576	0.95279
Pb^2+^	Lignite	0.01251	11.85742	0.96693	0.00038	19.03300	0.79657	0.47558	0.33625	0.91686	1.47048	− 3.53485	0.98318
	MML	0.01327	14.01561	0.98903	0.00052	19.59630	0.99463	0.63466	0.29240	0.94079	1.63444	− 3.37912	0.97145

From Fig. 4a,b, it can be seen that the tangent slope of the curve is larger at the beginning of the adsorption, indicating that the adsorption rate of MML and lignite to adsorb heavy metal ions is faster. Then the slope gradually decreases and the adsorption rate decreases. This is because there are enough effective adsorption sites on the surface of the adsorbent at the initial stage. As the reaction progresses, the adsorption sites are gradually occupied, resulting in the reduction of the adsorption efficiency. On the other hand, the experimental results (Fig. 4a) show that for the single-component mode, as reported by Jellali et al.^18^, the adsorption efficiency of the studied heavy metal ions by lignite is as follows: Pb^2+^ > Cu^2+^ > Zn^2+^. However, for the single-component mode, the adsorption efficiency of the studied heavy metal ions by MML is as follows: Pb^2+^ > Zn^2+^ > Cu^2+^ (Fig. 4b). This may be because the magnetic modification affected the physico-chemical properties of the lignite, such as the donor atoms abundance (oxygen, nitrogen, sulfur)^40^.

As can be seen from Table 2, the Quasi first-order kinetic parameters *R*^*2*^ for the adsorption of Cu^2+^, Zn^2+^ and Pb^2+^ by lignite are higher (*R*^*2*^ > 0.96), indicating that the adsorption process followe the Quasi first-order kinetic model and is dominated by physisorption^41^. The fitted equations of the Quasi first-order kinetics model of lignite for Cu^2+^, Zn^2+^ and Pb^2+^ are: y = 9.2602*(1 − e^−0.00607x^), y = 10.2839*(1 − e^−0.00468x^), and y = 11.8456*(1 − e^−0.01265x^), respectively. In contrast, the Quasi second-order kinetic parameter *R*^*2*^ for the adsorption of heavy metal ions by MML is higher than that of the Quasi first-order kinetic, indicating that the Quasi second-order kinetic model fits well the experimental data for three heavy metal ions^25^. Moreover, the Quasi second-order kinetic equilibrium adsorption capacity is closer to the experimental adsorption capacity. Therefore, under the used experimental conditions, the Quasi second-order kinetic model is more suitable for fitting the adsorption process of Cu^2+^, Zn^2+^ and Pb^2+^ by MML. The Quasi second-order kinetic model shows that the adsorption process of the studied heavy metal ions by MML is mainly chemisorption, and the adsorption rate is affected by the coordination between the surface active site of adsorbent and the heavy metal ions^42^. The fitted equations for the Quasi second-order kinetics model of MML for Cu^2+^, Zn^2+^ and Pb^2+^ are: y = 0.09599x + 8.81861, y = 0.09333x + 10.01582, and y = 0.05103x + 5.02836, respectively.

#### Elovich model

The Elovich model assumes that with the increase of the amount of heavy metal ions, the adsorption rate decreases exponentially, following a chemisorption mechanism. The model is expressed in the following form^43^:

6 $${q}_{t}=\frac{1}{b}\mathrm{ln}\left(ab\right)+\frac{1}{b}\mathrm{ln}\,t$$

where a and b are Elovich constants.

The experimental data were fitted by the Elovich model, and the results were shown in Fig. 4 and Table 2. As can be seen, Elovich model is in good agreement with the experimental data of adsorption of Cu^2+^, Zn^2+^ and Pb^2+^ by lignite and MML, and *R*^*2*^ is between 0.87 and 0.97 (Fig. 4e,f). It shows that there is chemisorption between adsorbents (lignite and MML) and three kinds of heavy metal ions.

#### Intra-particle diffusion model

The adsorption process usually involves two main mechanisms: film diffusion and particle diffusion. In order to determine the way of metal ions entering the adsorbent material from the solution, the Intra -particle diffusion model (Eq. 7) was used to determine the adsorption rate control steps and the results are shown in Fig. 4 and Table 2^44^.

7 $${q}_{t}={k}_{3}{t}^{1/2}+C$$

where *q*_*t*_ is the amount of metal ions adsorbed at any moment *t* (mg/g), *k*_*3*_ is the diffusion rate constant within the particle (min^−1^), and *C* is the constant involving thickness and boundary layer. The larger the value of *C*, the greater the contribution of the boundary layer.

Figure 4g,h show the linear relationship between *q*_*t*_ and *t*^*1/2*^. Among them, the parameters of the Intra-particle diffusion model for Cu^2+^, Zn^2+^ and Pb^2+^ adsorption by lignite and MML were shown in Table 2. According to reports, if the plots are linear and pass through the origin, indicating that Intra-particle diffusion is the only rate control step; if the linear plot of the fitted results does not pass through the origin, indicating that the adsorption rate is also controlled by other adsorption stages^45^. As can be seen in Fig. 4, the fitted results for the adsorption of Cu^2+^, Zn^2+^ and Pb^2+^ by lignite and MML are linear and do not pass the origin, indicating that the rates of Cu^2+^, Zn^2+^ and Pb^2+^ adsorption by lignite and MML are jointly controlled by multiple adsorption stages. In addition, linearity indicates spontaneous utilization of available adsorption sites on adsorbent surfaces^38^.

### Adsorption thermodynamics

Based on the adsorption experimental data affected by temperature, the thermodynamic parameters (Gibbs free energy changes, *ΔG*; entropy, *ΔS* and enthalpy change, *ΔH*) determined in the following forms^37^:

8 $$K=\frac{{q}_{e}}{{c}_{e}}$$

9 $$\Delta G=-RT\,\mathrm{ln}\,K$$

10 $$\mathrm{ln}K=\frac{\Delta S}{R}-\frac{\Delta H}{RT}$$

where *R* is the ideal gas constant (8.314 J/mol·K), *T* is the Kelvin temperature (K), and *K* is the thermodynamic equilibrium constant.

**Figure 5:**
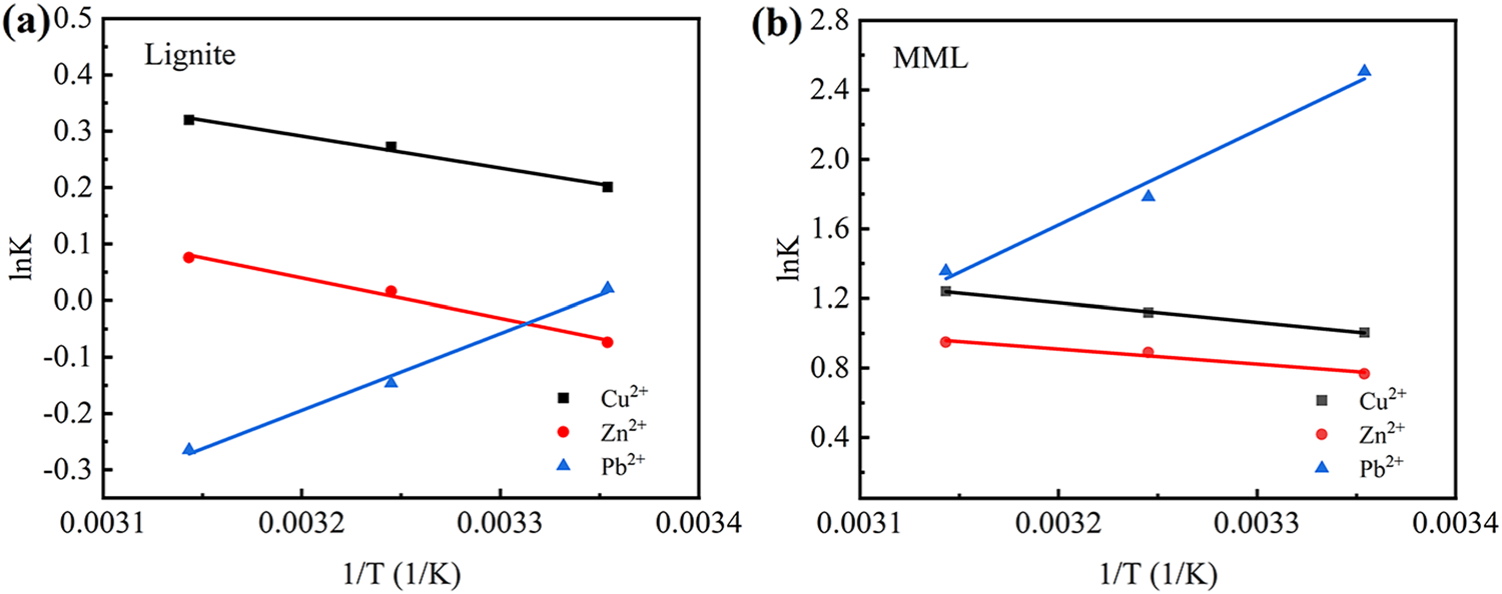
Relationship curve between lnK and 1/T: (**a**) lignite; (**b**) MML.

According to the experimental results, the thermodynamic parameters were determined, as shown in Fig. 5 and Table 3. The positive *ΔH* values of Cu^2+^ and Zn^2+^ indicates that the adsorption of Cu^2+^ and Zn^2+^ by lignite and MML is endothermic adsorption, while the negative *ΔH* values of Pb^2+^ indicates that the adsorption is exothermic adsorption. With the increase of temperature, the negative *ΔG* values of Cu^2+^ and Zn^2+^ become more negative, which indicates that the adsorption efficiency is higher at higher temperature and the adsorption is spontaneous. The negative *ΔG* values of Pb^2+^ tend to positive, indicating that the adsorption is spontaneous but its adsorption efficiency is lower at higher temperature. The results show that the temperature increase is beneficial to the adsorption of Cu^2+^ and Zn^2+^ by lignite and MML, but not conducive to the adsorption of Pb^2+^. In addition, the *ΔG* values of an adsorbent for the adsorption of some heavy metal ions at different temperatures are very close, which indicates that the adsorption process of Cu^2+^, Zn^2+^ and Pb^2+^ by lignite and MML is not obviously affected by temperature.

**Table 3:** Thermodynamic parameters for adsorption of Cu^2+^, Zn^2+^ and Pb^2+^ onto different samples: Lignite; MML.

Adsorption material	Metal ion	ΔH (kJ/mol)	ΔS (J/mol K)	ΔG (kJ/mol)		
				298.15 K	308.15 K	318.15 K
Lignite	Cu^2+^	4.70583	17.48052	− 0.49799	− 0.69788	− 0.846643
	Zn^2+^	5.92687	19.29696	0.18368	− 0.04125	− 0.20076
	Pb^2+^	− 11.30457	− 37.79145	− 0.05280	0.37456	0.70069
MML	Cu^2+^	9.26270	39.40279	− 2.49072	− 2.86760	− 3.27939
	Zn^2+^	7.19446	30.58529	− 1.90175	− 2.27912	− 2.51020
	Pb^2+^	− 45.2219	− 131.86095	− 6.21069	− 4.56951	− 3.58834

### Desorption

**Figure 6:**
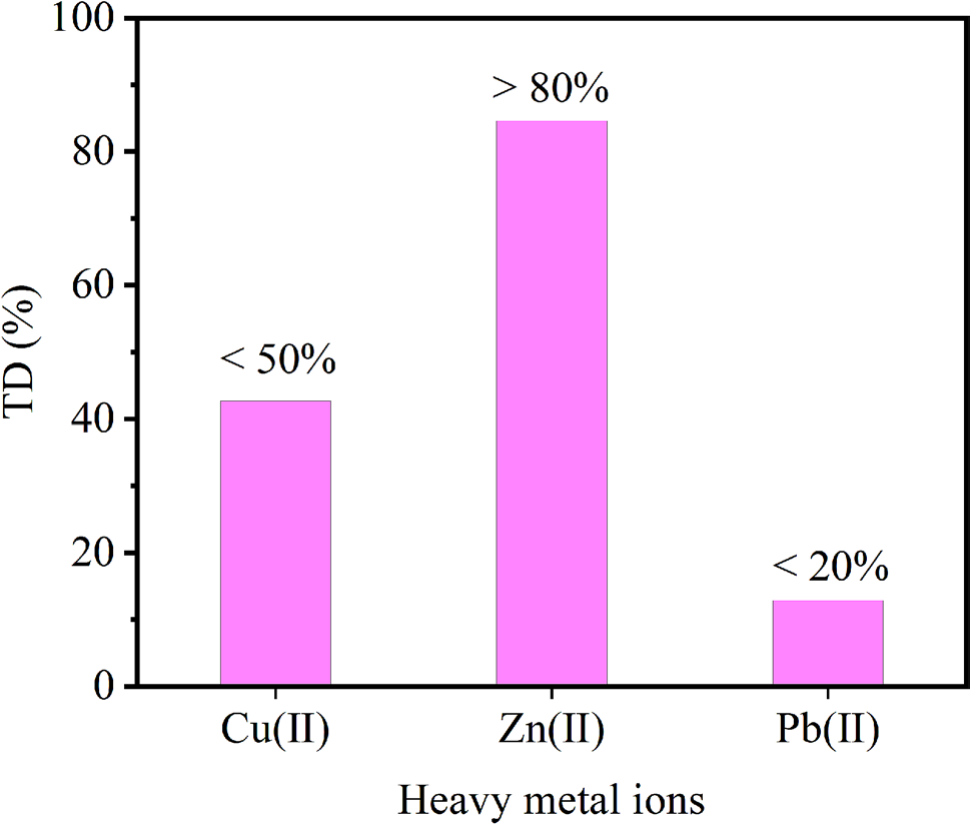
Desorption rate of heavy metal ions by MML.

To clarify the properties of MML adsorption of Cu^2+^, Zn^2+^ and Pb^2+^, desorption experiments were used to analyze the adsorption process. The desorption agents commonly used for desorption of adsorbents are NaOH, HCl, HNO_3_, EDTA, CaCl_2_ and organic solvents such as methanol and ethanol^46^. In this study, 0.1 mol/L H_2_SO_4_ was used as desorption agent for desorption experiment. Figure 6 shows that the desorption rates of Cu^2+^ and Pb^2+^ are less than 50%, indicating that the retention of MML for both metals is very strong under acidic conditions. Therefore, the adsorption process of Cu^2+^ and Pb^2+^ by MML is chemisorption. This result is consistent with the adsorption kinetics. However, the desorption rate of Pb^2+^ is as high as 84.62%, which may be due to the removal of Zn^2+^ in the form of Zn(OH)_2_ in solution. When the pH value of the desorption solution is low, the precipitate of Zn(OH)_2_ adsorbed on the surface of MML is dissolved, and Zn^2+^ re-enter the solution.

### Dynamic experimental analysis

**Figure 7:**
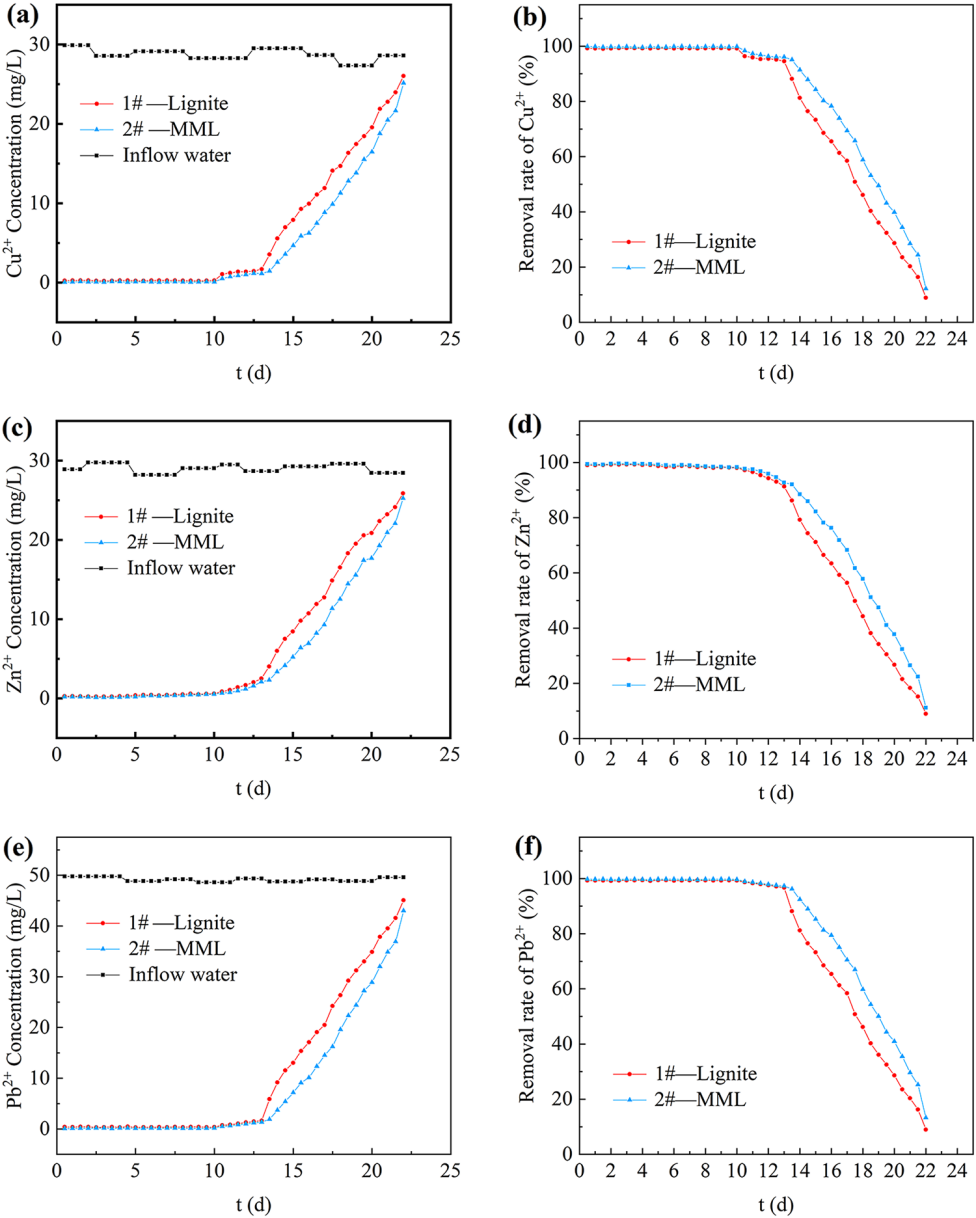
Removal effect of heavy metal ion. (**a**) Cu^2+^ concentration of effluent; (**b**) removal rate of Cu^2+^; (**c**) Zn^2+^ concentration of effluent; (**d**) removal rate of Zn^2+^; (**e**) Pb^2+^ concentration of effluent; (**f**) removal rate of Pb^2+^.

The dynamic removal effects of lignite and MML on Cu^2+^, Zn^2+^ and Pb^2+^ with time were shown in Fig. 7. The dynamic removal effects of both lignite and MML on Cu^2+^, Zn^2+^ and Pb^2+^ shows a similar trend (Fig. 7). Heavy metal ions are removed rapidly in the first 13 days, with removal rates of Cu^2+^, Zn^2+^ and Pb^2+^ exceeding 95, 92 and 97%, respectively. Then the removal rates gradually decreases from the 13th day to the 22nd day, and the removal rates is only about 10% at the 22nd day. This phenomenon is attributed to the fact that there are enough binding sites on the surfaces of adsorbent lignite and MML for metal ions to occupy at the initial stage, which make the adsorption process easier. However, the number of effective adsorption sites on the surfaces of lignite and MML gradually consumes with time, resulting in a decrease in the removal rates. During the whole dynamic removal cycle, the average removal rates of Cu^2+^, Zn^2+^ and Pb^2+^ by lignite are 78.00, 76.97 and 78.65%, respectively, and the average removal rates of the studied heavy metal ions by MML are 82.83, 81.57 and 83.50%, respectively. Apparently, magnetic modification increases the adsorption capacity of heavy metal ions by the lignite. This may be because the surface of MML is loaded with Fe_3_O_4_ particles, which increases the specific surface area of lignite^23^. In addition, the adsorption capacity of the three heavy metal ions in AMD by lignite and MML is as follows: Pb^2+^ > Cu^2+^ > Zn^2+^, which is the same as the result reported by Jellali et al.^18^.

### Characterization analysis

#### SEM analysis

**Figure 8:**
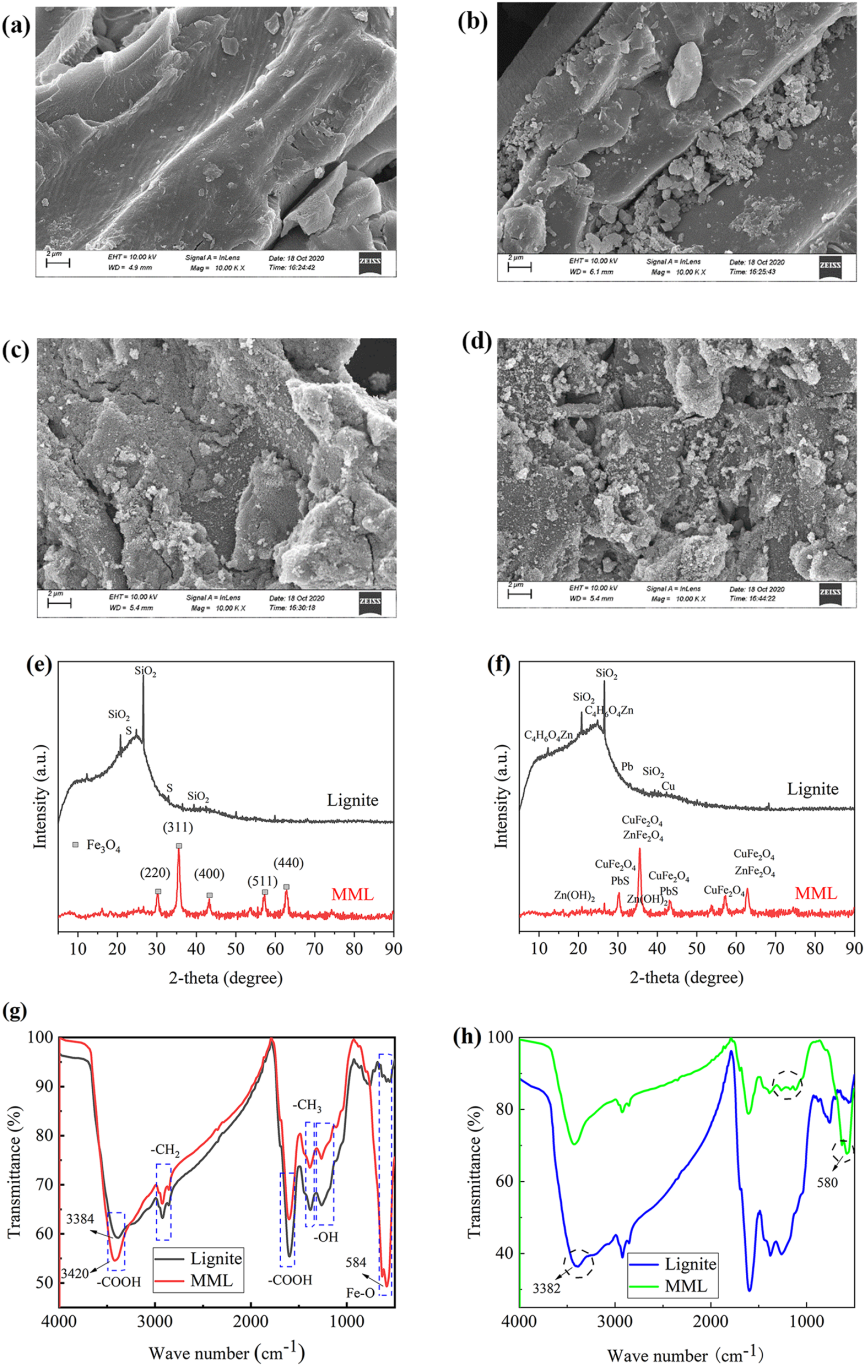
Characterization for adsorption of Cu^2+^, Zn^2+^ and Pb^2+^ onto different samples: lignite; MML. SEM images for before adsorption: (**a**) lignite; (**b**) MML. SEM images for after adsorption: (**c**) lignite; (**d**) MML. XRD patterns of lignite and MML: (**e**) before adsorption; (**f**) after adsorption. FTIR spectra of lignite and MML: (**g**) before adsorption, (**h**) after adsorption.

SEM detection results of lignite and MML before and after the dynamic test are shown in Fig. 8. From Fig. 8a,b, it appears that the raw lignite presents a smooth and porous surface with a lager pore size. However, MML presents a slightly rough surface, which is mainly because the successful loading of Fe_3_O_4_ on the lignite surface. A large number of Fe_3_O_4_ particles were scattered on the surface of lignite, which increased the specific surface area of the lignite and facilitated the removal of Cu^2+^, Zn^2+^ and Pb^2+^ by MML. From Fig. 8c, d, surface voids of lignite after adsorption of Cu^2+^, Zn^2+^ and Pb^2+^ are filled. Compared with lignite, the granular material on the surface of MML was significantly increased, indicating that more sediment was generated on the surface of MML.

#### XRD analysis

The XRD test results of lignite and MML before dynamic test are shown in Fig. 8e. The XRD patterns of lignite have relatively wide diffraction peaks (Fig. 8e). The diffraction peaks at 2θ = 20.6°, 26.70°, 39.5° and 2θ = 23.1°, 33.0° were caused by SiO_2_ and S in lignite, respectively. Compared with lignite, the number of wide peaks in MML XRD pattern decreased, and the characteristic peaks of SiO_2_ and S disappeared. New diffraction peaks appeared at 2θ = 30.09°, 35.42°, 43.05°, 56.93° and 62.52°, showing the diffraction planes of (220), (311), (400), (511) and (440). These diffraction planes are consistent with the standard XRD data of cubic Fe_3_O_4_, and it can be inferred that Fe_3_O_4_ is successfully deposited on the surface of lignite^24^. The significant surface phase changes are consistent with SEM, further verifying that the surface of MML became rougher due to the presence of Fe_3_O_4_. The rough surface of MML led to an increase in specific surface area, which is conducive to the adsorption of heavy metal ions.

The XRD test results of lignite and MML after dynamic test are shown in Fig. 8f. By comparing the XRD patterns of lignite and MML before and after the dynamic test, new diffraction peaks appeared at 2θ = 11.3°, 11.8°, 19.5° and 22.5° after the dynamic test, corresponding to C_4_H_6_O_4_Zn diffraction. New diffraction peaks appeared at 2θ = 31.4° and 43.2°, corresponding to Pb and Cu elemental diffraction, respectively. C_4_H_6_O_4_Zn was a metal compound formed by electrostatic interaction and coordination between Zn^2+^ and functional groups in humic acid. Cu and Pb were generated by the reduction of Cu^2+^ and Pb^2+^, which indicates that the adsorption of Cu^2+^, Zn^2+^ and Pb^2+^ by lignite is chemisorption. Combined with the analysis results of adsorption kinetics, the adsorption process of heavy metal ions by lignite involves physisorption and chemisorption, but mainly physisorption. After the reaction, new diffraction peaks appeared at 2θ = 30.1°, 35.5°, 43.1°, 57.0° and 62.6°, corresponding to the crystal plane diffraction of CuFe_2_O_4_, and new diffraction peaks appeared at 2θ = 35.3° and 62.3°, corresponding to the crystal plane diffraction of ZnFe_2_O_4_. It shows that Cu^2+^ and Zn^2+^ in AMD solution co-precipitated with Fe_3_O_4_, and the resulting sediments were attached to the surface of MML in the form of CuFe_2_O_4_ and ZnFe_2_O_4_. New diffraction peaks appeared at 2θ = 20.2° and 37.7°, corresponding to the crystal surface diffraction of Zn(OH)_2_. The occurrence of Zn(OH)_2_ may be due to the increase of pH value caused by the consumption of H^+^ in the reaction process. In addition, PbS diffraction peaks appeared at 2θ = 30.1° and 43.1°. This may be due to the fact that lignite contains a certain amount of S^2−^, which is dissolved and released as the reaction progresses and reacted with the free Pb^2+^ in AMD. The results show that MML can remove Pb^2+^ and S^2−^ at the same time, and prevent the oxidation of S^2−^ in lignite to SO_4_^2−^, causing secondary pollution. The appearance of phases such as CuFe_2_O_4_, ZnFe_2_O_4_, Zn(OH)_2_ and PbS confirms that the adsorption process of Cu^2+^, Zn^2+^ and Pb^2+^ by MML is mainly chemisorption.

#### FTIR analysis

The lignite and MML before and after the dynamic test were taken for FTIR detection, and the results are shown in Fig. 8g,h. The peaks of lignite and MML at 3400 cm^−1^ was caused by O–H stretching vibrations of carboxylic acid groups, and the peak at 2920 cm^−1^ was attributed to the presence of -CH_2_ in the stretching of aliphatic compounds, and the peak at 1600 cm^−1^ was related to the stretching vibrations of carboxylic acid functional groups^47^ (Fig. 8f, h). Some peaks of lignite changed after magnetic modification, especially the generation of new peaks at 584 cm^−1^, was attributed to Fe–O stretching vibrations of Fe_3_O_4_ particles^48^. It shows that Fe_3_O_4_ was successfully loaded onto the lignite surface, which is consistent with the XRD results.

After the dynamic test, the peak position and intensity of some functional groups in lignite changed slightly. For instance, the peaks value at 3384, 2921 and 1599 cm^−1^ moved to 3392, 2923 and 1597 cm^−1^, respectively. It shows that the adsorption of Cu^2+^, Zn^2+^ and Pb^2+^ by lignite is due to physisorption under van der Waals forces^28^. The peak value of MML at 586 cm^−1^ shifted to 575 cm^−1^, indicating the possibility of Fe–O combining with Cu^2+^ and Zn^2+^ through Fe–O–Cu and Fe–O–Zn. In addition, the peak shape of the hydroxyl group corresponding to 1111–1270 cm^−1^ also changed, which may be due to the reaction of Zn^2+^ and OH^−^ to produce Zn(OH)_2_ precipitation. These phenomena are consistent with XRD results. The stretching vibration of Zn^2+^ by hydroxyl group of carboxylic acid group in lignite generated metal compound C_4_H_6_O_4_Zn, and the stretching vibration of Cu^2+^ and Zn^2+^ by Fe–O of Fe_3_O_4_ particles in MML generated CuFe_2_O_4_ and ZnFe_2_O_4_.

### Compare the removal of Cu^2+^, Zn^2+^ and Pb^2+^ by different adsorbents

Although the Langmuir model assumes that monolayer adsorption occurs on the uniform adsorbent surface and there is no interaction between adsorbates, many researchers have used Langmuir constant *q*_*m*_ to evaluate the adsorption capacity of adsorbents. To compare the maximum adsorption capacity of Cu^2+^, Zn^2+^ and Pb^2+^ during the adsorption process, various adsorbents for Cu^2+^, Zn^2+^ and Pb^2+^ removal were prepared, as shown in Table 4. It shows that lignite and MML have higher adsorption capacity in removing Cu^2+^, Zn^2+^ and Pb^2+^. The maximum monolayer adsorption capacity of lignite are13.3618, 14.7929 and 17.5274 mg/g, respectively, and the maximum monolayer adsorption capacities of MML for Cu^2+^, Zn^2+^ and Pb^2+^ are 16.2127, 15.8053 and 18.3870 mg/g, respectively. This indicates that MML is a potential adsorbent for Cu^2+^, Zn^2+^ and Pb^2+^ adsorption from AMD. On the other hand, the optimum amount (4 g/L) of MML is lower than that of other sorbents. It shows that MML can be used as a low-cost adsorbent to remove Cu^2+^, Zn^2+^ and Pb^2+^ from AMD (Table 4).

**Table 4:** Comparison of the maximum monolayer adsorption capacities of Cu^2+^, Zn^2+^ and Pb^2+^ on various adsorbents.

Heavy metal	Adsorbent	Does (g/L)	Adsorption capacity (mg/g)	References
Cu^2+^	Hydroxyapatite	10.00000	10.58000	^49^
	Attapulgite/(La + Fe)	8.00000	7.15610	^50^
	Sewage sludge activated carbon	4.00000	4.04000	^51^
	Lignite	4.00000	13.36180	This work
	MML		16.21270	
Zn^2+^	Red earth	10.00000	8.74000	^52^
	Functionalized wool	5.00000	1.09000	^53^
	Sugarcane-bagasse ash	10.00000	3.34798	^54^
	Lignite	4.00000	14.79290	This work
	MML		15.80530	
Pb^2+^	Carbonised sugarcane bagasse	10.00000	7.29930	^55^
	Attapulgite/(La + Fe)	8.00000	4.00270	^50^
	Mangrove bark (Rhizopora mucronata)	16.00000	18.28100	^56^
	Lignite	4.00000	17.52740	This work
	MML		18.38700	

## Conclusion

1. The best adsorption conditions for Cu^2+^, Zn^2+^ and Pb^2+^ by lignite and MML were pH = 4, adsorbent dosage 4 g/L and temperature 25℃. Under the same metal concentration conditions, the adsorption capacity and removal rates of Cu^2+^, Zn^2+^ and Pb^2+^ by MML were higher than that of lignite. When the concentration of heavy metal ions in AMD solution was higher, MML had greater adsorption potential than lignite.
2. The isothermal adsorption of Cu^2+^, Zn^2+^ and Pb^2+^ by lignite and MML was consistent with the Langmuir model, indicating that the adsorption was consistent with the monolayer layer adsorption process. The adsorption processes of Cu^2+^, Zn^2+^ and Pb^2+^ by lignite obeyed the Quasi first-order kinetic model, indicating that the adsorption process was dominated by physisorption, and the fitted equations were: y = 9.2602*(1 − e^−0.00607x^), y = 10.2839*(1 − e^−0.00468x^), and y = 11.8456*(1 − e^−0.01265x^), respectively. The adsorption process of MML obeyed the Quasi second-order kinetic model, which indicates that the adsorption process was dominated by chemisorption and the adsorption rate was affected by the coordination between the surface active site of adsorbent and the heavy metal ions, and the fitted equations are: y = 0.09599x + 8.81861, y = 0.09333x + 10.01582, y = 0.05103x + 5.02836. The fitting results of Intra-particle diffusion model showed that the adsorption processes of lignite and MML was jointly controlled by multiple adsorption stages. The Elovich model and desorption experiments confirmed that the adsorption process of Cu^2+^, Zn^2+^ and Pb^2+^ by MML was mainly chemisorption. The adsorption thermodynamics showed that the adsorption of Cu^2+^ and Zn^2+^ by lignite and MML was spontaneous and endothermic, while the adsorption of Pb^2+^ was exothermic.
3. The dynamic experimental results showed that the removal effect of heavy metal ions by lignite was significantly better than that of lignite. The average removal rates of Cu^2+^, Zn^2+^ and Pb^2+^ by lignite were 78.00, 76.97 and 78.65%, respectively, and the average removal rates of the studied heavy metal ions by MML were 82.83, 81.57 and 83.50%, respectively. In addition, the adsorption capacity of the three heavy metal ions in AMD by lignite and MML is as follows: Pb^2+^ > Cu^2+^ > Zn^2+^.
4. From SEM, XRD and FTIR tests, it showed that Fe_3_O_4_ was successfully loaded onto the lignite surface during the magnetic modification process. SEM test showed that the surface morphology of lignite was rougher after magnetic modification, and more sediment was generated on MML surface after the reaction. XRD results showed that Lignite and MML removed Cu^2+^, Zn^2+^ and Pb^2+^ from AMD in different forms. FTIR results showed that the adsorption process of Cu^2+^, Zn^2+^ and Pb^2+^ by MML was related to the O–H stretching vibration of carboxylic acid ions and Fe–O stretching vibration of Fe_3_O_4_ particles.
5. The raw lignite has the characteristics of low cost and wide source. Using magnetic modification method to modify lignite can not only improve the adsorption capacity of the lignite, but also solve the problem that the lignite is difficult to separate from the solution. In this paper, a new modification method of lignite was proposed, which verified the feasibility of MML in the treatment of AMD, and provided a basis for the adsorption and use of MML.

## Materials and methods

### Materials and chemicals

The lignite was purchased from Shanxi Fuhong Mineral Products Co., Ltd. FeSO_4_·7H_2_O, HNO_3_, Fe_2_(SO_4_)_3_, NH_3_·H_2_O, CuSO_4_·5H_2_O, ZnSO4·7H_2_O, Pb(NO_3_)_2_, Na_2_SO_4_, H_2_SO_4_ were purchased from Liaoning Quanrui Reagent Co., LTD. Chemicals and reagents were analytical grade.

### Adsorbent preparation

Lignite: Lignite was pulverized with a high-speed mill and screened out with a diameter of 250 mesh (58 μm). The lignite was immersed in deionized water for 2–3 times to remove impurities, and then dried at 60 °C for 24 h as the raw material.

MML: The chemical co-precipitation method is used to magnetically modify the lignite, that is, to load Fe_3_O_4_ magnetic particles on the surface of the lignite^57^. The formation reaction formula of Fe_3_O_4_ is as follows.

11 $${\text{Fe}}^{2+}\text{+}{\text{Fe}}^{3+}+ \text{8} {\text{OH}}^{-}={\text{Fe}}_{3}{{\text{O}}}_{4}{ \downarrow +4}{\text{H}}_{2}{\text{O}}$$

**Figure 9:**
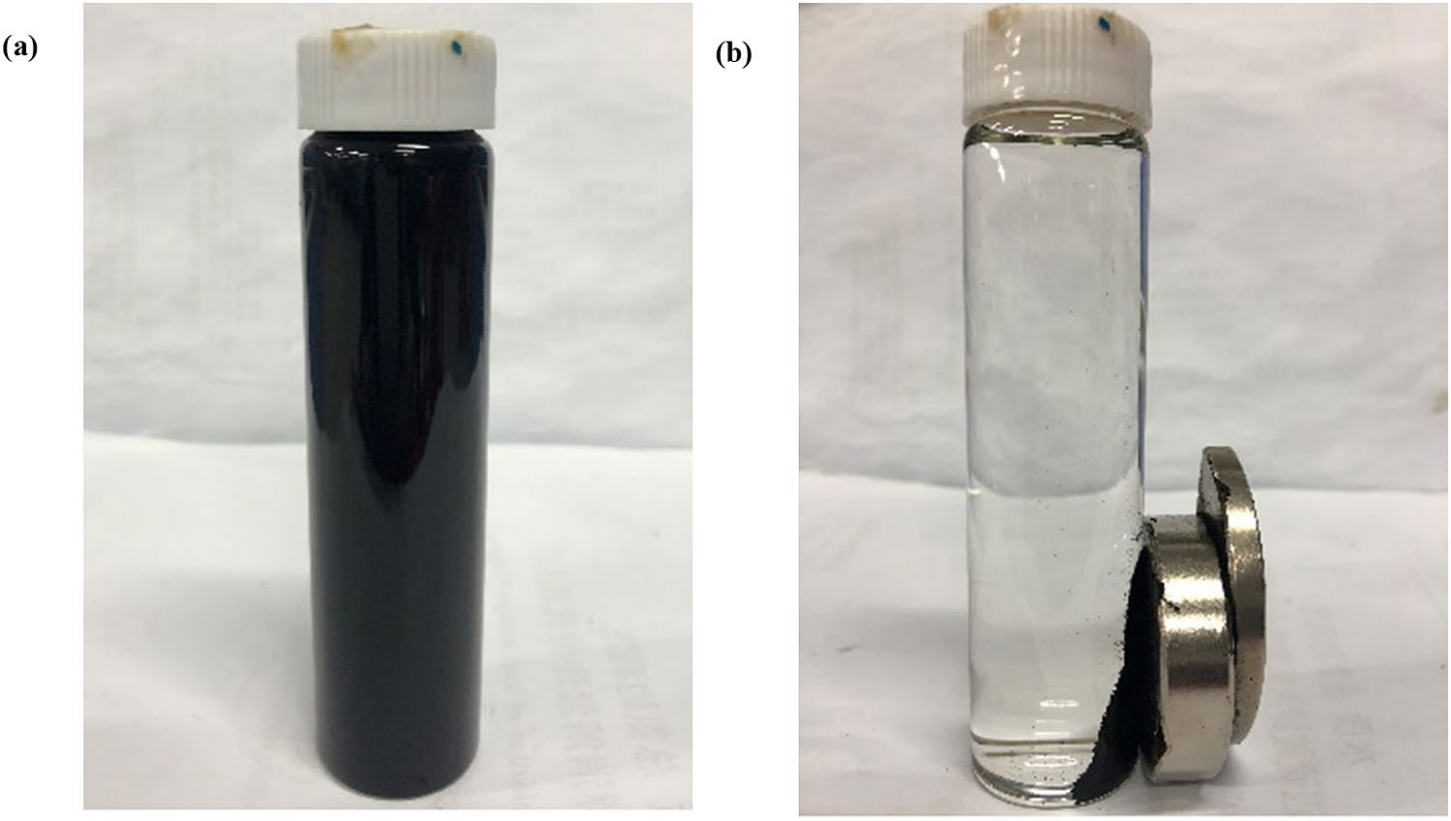
Comparison of solid–liquid separation effects between lignite and MML. (**a**) Lignite; (**b**) MML.

The molar ratio of Fe^3+^ to Fe^2+^ substance was set to 2:1 (i.e. 1.31 g FeSO_4_·7H_2_O and 1.88 g Fe_2_(SO_4_)_3_). 200 mL and 0.7 mol/L iron ion solution was prepared and placed in a thermostatic water bath at 60 °C. Weigh 10 g lignite and add it to iron solution, stir it for 1 h under the action of an electric stirrer with speed regulation at 350r/min, then add concentrated ammonia water with mass fraction of 25% drop by drop to pH value of 9, continue to stir for 1 h, and stand for 2 h for aging. The resulting precipitate was repeatedly cleaned with deionized water to make the supernatant neutral and then separated by magnets to obtain the magnetic material. The magnetic material was dried in a vacuum drying oven for 12 h to obtain MML. The comparison of solid–liquid separation between lignite and MML in Fig. 9.

### Heavy metal ions solutions preparation and analysis

CuSO_4_·5H_2_O、ZnSO_4_·7H_2_O and Pb(NO_3_)_2_ were used to prepare Cu^2+^, Zn^2+^ and Pb^2+^ standard solutions, respectively. Metal concentrations were measured thorough an atomic absorption spectrometer (AAS) with an air-acetylene flame (Hitachi-Z2000, Japan). The wavelengths used for the analysis of the Cu^2+^, Zn^2+^, and Pb^2+^ were 324.8, 213.9, and 283.3 nm, respectively. The pH values of the solutions were adjusted by using 3% nitric acid or sodium hydroxide.

### Experimental methods

#### Adsorption condition optimization experiment

Prepared a Cu^2+^ standard solution with a concentration of 30 mg/L. Added a certain amount of lignite and MML into 250 mL conical flasks containing 250 mL Cu^2+^ standard solution. Placed the conical flasks in a constant tremors shaking at 150 r/min, adsorb for 180 min, and then sample with a pipette gun. The samples were filtered through a 0.45 μm microporous membrane before analysis with AAS. Calculated the removal rates and adsorption amounts of heavy metal ions by Eqs. (12) and (13)^58^. All the experiments were repeated three times, and the average values were taken as the final measured values. The research was carried out by changing the pH value of the solution (2–4), adsorbent dose (2–6 g/L) and temperature (298.15, 308.15 and 318.15 K). The test conditions and process of the Zn^2+^ standard solution with a concentration of 30 mg/L and the Pb^2+^ standard solution with a concentration of 50 mg/L were exactly the same as the Cu^2+^ adsorption test.

12 $$E=\frac{{C}_{0}-{C}_{t}}{{C}_{0}}\times 100\%$$

13 $${q}_{e}=\frac{\left({C}_{0}-{C}_{e}\right)V}{M}$$

where *E* is the removal rate (%), *C*_*0*_ is the initial mass concentration (mg/L), *C*_*t*_ is the mass concentration of the remaining metal ions in the solution at time t (mg/L), *q*_*e*_ is the adsorption amount at equilibrium (mg/g), *C*_*e*_ is the mass concentration of the remaining metal ions in the solution at equilibrium (mg/L), *V* is the volume of the solution (L), and *M* is the mass of the adsorbent material (g).

#### Adsorption isotherm experiment

Prepared Cu^2+^, Zn^2+^ and Pb^2+^ solutions with initial concentrations of 10, 30, 50, 70 and 90 mg/L, respectively, and adjusted the pH values of each solution to 4. Taken 250 mL of Cu^2+^, Zn^2+^ and Pb^2+^ solutions with different concentrations in a 250 mL conical flask, and added 1 g lignite or MML into the solutions. Placed the conical flasks in a constant tremors shaking at 25 °C, 150 r/min, absorbed for 180 min, and then sampled with a pipette gun. The samples were filtered through a 0.45 μm microporous membrane before analysis with AAS. Calculated the removal rates and adsorption amounts of heavy metal ions by Eqs. (12) and (13). All the experiments were repeated three times, and the average values were taken as the final measured values.

#### Adsorption kinetics experiment

Prepared a Cu^2+^ standard solution with a concentration of 30 mg/L and adjust the pH value to 4. Weighed 1 g each of lignite and MML, and added them to two 250 mL conical flasks containing 250 mL of 30 mg/L Cu^2+^ standard solution. Placed the conical flasks in a constant tremors shaking at 25 °C and 150 r/min to desorb. At intervals (5, 10, 15, 30, 60, 90, 120 and 180 min), measured the Cu^2+^ concentrations thorough AAS. Calculated the removal rates and adsorption amounts. The test conditions and process of the Zn^2+^ standard solution with a concentration of 30 mg/L and the Pb^2+^ standard solution with a concentration of 50 mg/L were exactly the same as the Cu^2+^ adsorption test. All the experiments were repeated three times and the average values were taken as the final measured values.

#### Adsorption thermodynamics experiment

Prepared a Cu^2+^ standard solution with a concentration of 30 mg/L and adjust the pH value to 4. Weighed 1 g of lignite or MML and added it to a 250 mL conical flask containing 250 mL of 30 mg/L Cu^2+^ standard solution. Placed the conical flasks in constant tremors shaking at 150 r/min for 180 min in different temperature (298.15, 308.15 and 318.15 K), and then sample with a pipette gun. The samples were filtered through a 0.45 μm microporous membrane before analysis with AAS. Calculated the removal rates and adsorption amounts of heavy metal ions by Eqs. (12) and (13). The test conditions and process of the Zn^2+^ standard solution with a concentration of 30 mg/L and the Pb^2+^ standard solution with a concentration of 50 mg/L were exactly the same as the Cu^2+^ adsorption test. All the experiments were repeated three times, and the average values were taken as the final measured values.

#### Desorption experiment

Prepared a Cu^2+^ standard solution with a concentration of 30 mg/L and adjust the pH value to 4. Weighed 1 g of MML and added it to a 250 mL conical flask containing 250 mL of 30 mg/L Cu^2+^ standard solution. Placed the conical flasks in a constant tremors shaking at 25 °C and 150 r/min to oscillate and react for 180 min before analysis with AAS. Used a magnet to take out the reacted MML. The collected MML was washed several times with deionized water and then added to a 250 mL conical flask containing 250 mL of 0.1 mol/L H_2_SO_4_ solution. Placed the conical flasks in a constant tremors shaking at 25 °C and 150 r/min to desorb for 180 min before analysis with AAS. Calculated the desorption amounts and desorption rates by Eqs. (14) and (15)^59^. The test conditions and process of the Zn^2+^ standard solution with a concentration of 30 mg/L and the Pb^2+^ standard solution with a concentration of 50 mg/L were exactly the same as the Cu^2+^ adsorption test. All the experiments were repeated three times and the average values were taken as the final measured values.

14 $${q}_{d}=\frac{CV}{M}\times 100\%$$

15 $$TD=\frac{{q}_{d}}{{q}_{e}}\times 100\%$$

where *q*_*e*_ is the adsorption capacity at equilibrium (mg/g), *Q*_*d*_ is desorption amount (mg/g), *C* is the concentration of heavy metal ions in ethanol solution at 180 min (mg/L), *V* is the volume of solution (L), *M* is the mass of adsorption material (g), and TD is desorption rate (%). If TD > 50%, the adsorption is physical adsorption. If TD < 50%, chemisorption.

#### Dynamic experiment

**Figure 10:**
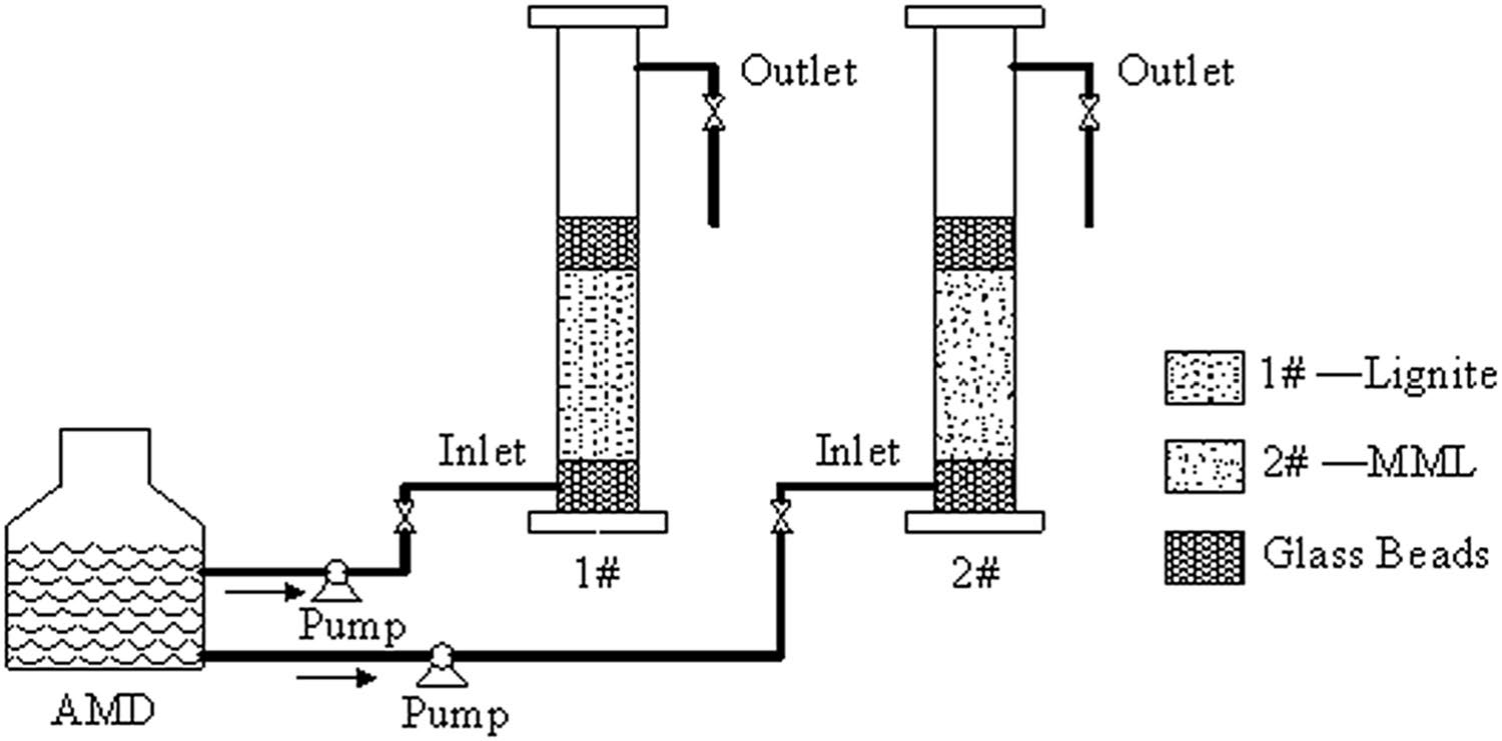
Dynamic test running device diagram.

Two Plexiglas’s tubes with an inner diameter of 40 mm and a height of 250 mm were filled with lignite and MML respectively. Glass beads with a height of 25 mm and a diameter of 3–5 mm were arranged at the top and botton of the dynamic column, and 100 mm high lignite was filled in the middle of #1 Plexiglas tube, and 100 mm high MML was filled in the middle of #2 Plexiglas tube. According to the results of the static beaker experiments, prepared the AMD with Cu^2+^ and Zn^2+^ concentration of 30 mg/L and Pb^2+^ concentration of 50 mg/L, and adjusted the pH value to 4. The overall operation mode adopted "bottom in and top out" continuous operation, and the inlet water flow rate was adjusted to 0.556 mL/min by peristaltic pump and flowmeter. The experimental device was shown in Fig. 10. The two groups of dynamic columns were operated at room temperature for 22 days, and samples were taken every 12 h. After the samples were filtered by 0.45 μm microporous membrane, the concentrations of Cu^2+^, Zn^2+^ and Pb^2+^ in the solution were determined thorough AAS.

### Adsorbent characterization

Lignite and MML before and after dynamic test were characterized by various techniques. SEM (Zeiss-Sigma 500, GER) was used to analyze the morphology and surface morphology of the adsorbent before and after adsorption. The phase and structure of the adsorbent were determined by XRD (Rigaku-Smartlab9, Japan). FTIR (Thermo Fisher-Nicolet iS5, USA) was recorded in the 500–4000 cm^−1^ range to study the surface functional groups before and after adsorption.

## Data Availability

All data generated or analyzed during this study are included in this published article.
